# Prevalence and Molecular Characterization of Human Bocavirus Detected in Croatian Children with Respiratory Infection

**DOI:** 10.3390/v13091728

**Published:** 2021-08-31

**Authors:** Sunčanica Ljubin-Sternak, Anamarija Slović, Maja Mijač, Mirna Jurković, Dubravko Forčić, Irena Ivković-Jureković, Tatjana Tot, Jasmina Vraneš

**Affiliations:** 1 Molecular Microbiology Department, Dr. Andrija Štampar Teaching Institute of Public Health, 10000 Zagreb, Croatia; maja.mijac@stampar.hr (M.M.); jasmina.vranes@stampar.hr (J.V.); 2 Medical Microbiology Department, University of Zagreb School of Medicine, 10000 Zagreb, Croatia; 3 Center of Excellence for Virus Immunology and Vaccines, Center for Research and Knowledge Transfer in Biotechnology, University of Zagreb, 10000 Zagreb, Croatia; aslovic@unizg.hr (A.S.); mirna.jurkovic@unizg.hr (M.J.); dforcic@unizg.hr (D.F.); 4 Department of Pulmonology, Allergy, Immunology and Rheumatology, Children’s Hospital Zagreb, 10000 Zagreb, Croatia; irena.ivkovicjurekovic@kdb.hr; 5 Faculty for Dental Medicine and Healthcare/School of Medicine, Josip Juraj Strossmayer University of Osijek, 31000 Osijek, Croatia; 6 Microbiology Department, General hospital Karlovac, Karlovac, 47000 Karlovac, Croatia; tatjanatotka@gmail.com

**Keywords:** bocavirus, respiratory tract, prevalence, Croatia, phylogeny, recombination

## Abstract

Human bocavirus (HBoV) 1 is considered an important respiratory pathogen, while the role of HBoV2-4 in clinical disease remains somewhat controversial. Since, they are characterized by a rapid evolution, worldwide surveillance of HBoVs’ genetics is necessary. This study explored the prevalence of HBoV genotypes in pediatric patients with respiratory tract infection in Croatia and studied their phylogeny. Using multiplex PCR for 15 respiratory viruses, we investigated 957 respiratory samples of children up to 18 years of age with respiratory tract infection obtained from May 2017 to March 2021 at two different hospitals in Croatia. Amplification of HBoV near-complete genome or three overlapping fragments was performed, sequenced, and their phylogenetic inferences constructed. HBoV was detected in 7.6% children with a median age of 1.36 years. Co-infection was observed in 82.2% samples. Sequencing was successfully performed on 29 HBoV positive samples, and all belonged to HBoV1. Croatian HBoV1 sequences are closely related to strains isolated worldwide, and no phylogenetic grouping based on mono- or co-infection cases or year of isolation was observed. Calculated rates of evolution for HBoV1 were 10^−4^ and 10^−5^ substitutions per site and year. Recombination was not detected among sequences from this study.

## 1. Introduction

Human bocavirus (HBoV) was discovered 16 years ago by Allander et.al. using a new molecular method for screening respiratory samples collected from children who had respiratory tract infection (RTI) of unknown etiology [1]. Additional serological and quantitative PCR research have provided compelling evidence that the newly discovered virus (later classified as HBoV1) acts as an etiologic agent for respiratory tract infections [2,3]. In the following years, three other genotypes (HBoV2-4) were discovered [4,5], primarily from the gastrointestinal tract; therefore, it seems to be involved in pathogenesis of gastroenteritis. Although HBoV2-4 has also been detected in samples from the respiratory tract but with a much lower frequency, their role in the pathogenesis of respiratory infections is unclear.

HBoVs are small viruses up to 26 nm in diameter, nonenveloped, DNA viruses that belong to the *Parvoviridae* family, *Bocaparvovirus* genus. Members of two species within *Bocaparvovirus* genus infect humans: HBoV1 and HBoV3 belonging to the species *Primate bocaparvovirus 1*, and HBoV 2 and HBoV 4 belonging to the species *Primate bocaparvovirus 2* [6]. HBoV possess a linear, single-stranded DNA genome of 5543 nucleotides (nt) long with non-identical terminal hairpins of 140 and 122 nt that play a key role in virus replication [7,8]. Recent advances in molecular biology research of HBoV1 revealed that during its replication in the polarized/nondividing airway epithelial cells HboV1 expresses six nonstructural proteins: NP1, NS1, NS1-70, NS2, NS3, and NS4, depending on splicing mRNAs within ORF 1 [9]. The mRNA spliced at the D2-A2 sites results in a shift of the NS1 ORF at the C-terminus and encodes NS1. Unspliced mRNA that reads the D2-A2 intron encodes the NS1-70 protein, and alternative splicing within the NS1-coding region generates mRNAs that encode NS2, NS3 and NS4 [9]. Moreover, HBoV1 also expresses viral non-coding RNA (BocaSR) and three structural proteins VP1, VP2, and VP3. The BocaSR is the first identified RNA polymerase III (Pol III) transcribed viral non-coding RNA in small DNA viruses [9].

HBoVs are considered to be highly diverse pathogens characterized by a rapid evolution [5,10]; therefore, worldwide surveillance of HBoVs’ genetic evolution is necessary. Although both mutation and recombination are responsible for bocavirus evolution, in a HBoV genome prone to rapid evolution with low level of polymorphisms, recombination seems to play a dominant role [5,11,12].

In this study, we focused on an investigation of the prevalence, phylogenetics, and evolution of HBoVs from pediatric patients with RTI, which will help to reveal the molecular epidemiology and phylogeny of the circulating HBoV in Croatia.

## 2. Materials and Methods

### 2.1. Patients and Sample Collection

Respiratory specimens obtained from 957 children up to 18 years of age were collected from May 2017 to March 2021. From each patient, nasopharyngeal and pharyngeal flocked swabs were collected, combined, and placed in viral transport medium (UTM™, Copan, Italy). Specimens were immediately transported to the molecular microbiology laboratory at the Public Health Institute, where they were stored at −80 °C until tested. Patients were recruited from two Croatian hospitals: Clinical Hospital Zagreb (*n* = 736) and General Hospital Karlovac (*n* = 221), respectively. Inclusion criteria were clinical diagnosis of acute respiratory tract infection (ARI) and need for hospitalization. Exclusion criteria were presumed to be bacterial respiratory infection according to biochemical parameters and healthcare-associated infection. Patients were categorized into the four groups according to age (<1, 1–2.99, 3–4.99 and ≥5 years of age), and two groups regarding the localization of infection (upper respiratory tract infection (URTI) and lower respiratory tract infection (LRTI)), according to the symptoms and signs previously described [13]. Demographic and clinical illness data were retrospectively analyzed by review of patient charts.

### 2.2. Respiratory Virus Detection

Specimens were tested for HBoVs and 14 other respiratory viruses using multiplex PCR. To isolate viral DNA and RNA from viral transport medium, 300 μL were extracted according to the manufacturer’s protocol using Ribospin™ vRD Kit (GeneAll Biotechnology, Seoul, Korea). During the first three years of the study, amplification of specific viral nucleic acid was performed using Seeplex^®^ RV15 detection kit (Seegene Inc., Seoul, Korea), followed by detection of PCR products by microchip electrophoresis on the MCE^®^-202 MultiNA device (Shimadzu, Kyoto, Japan) [13]. From mid-2020, multiplex-tandem polymerase chain reaction kit for respiratory viruses (MT-PCR, AusDiagnostics Pty Ltd., Sydney, NSW, Australia) was used, according to the manufacturer’s instructions [14]. Both assays detect the following viruses: rhinoviruses (RV), adenoviruses (AdV), seasonal coronaviruses 229E/NL63 and OC43 (HCoV), parainfluenza virus types 1–4 (PIV), respiratory syncytial virus types A and B (RSV), influenza virus types A and B (Flu), human metapneumovirus (HMPV), enterovirus (EV) and HBoV, but MT-PCR additionally detects human parechovirus and SARS-CoV-2.

### 2.3. Next Generation Sequencing

For next generation sequencing (NGS), viral DNA was re-extracted from 400 µL of positive samples using Quick-DNA Viral Kit (Zymo Research) following manufacturer’s recommendations. Isolated DNA was subjected to PCR amplification using Phusion polymerase (New England Biolabs), either by amplification of near-complete genome using primers B_fw and B_rev (covering positions 1–5212, according to sequence KP710213), or combinations of primers generating three overlapping fragments, respectively. Primer sequences and reaction conditions are shown in Supplementary File 1. Fragments were separated on 1% agarose gels, excised, and purified using Nucleospin Gel and PCR Clean-up Kit (Macherey-Nagel, Dueren, Germany) and quantified with QuantiFluor^®^ ONE dsDNA System (Promega, Madison, WI, USA). Libraries were prepared using NEBNext Ultra II FS DNA Library prep kit (New England Biolabs, Ipswich, Great Britain) quality checked on 2100 Bioanalyzer and High Sensitivity DNA Kit (Agilent, Santa Clara, CA, USA). Libraries were pooled and sequenced on MiniSeq Mid Output Kit (2 × 150 paired-end reads, Illumina, San Diego, CA, USA).

### 2.4. Sequence Analysis

Quality of raw reads was assessed with FastQC v0.11.8 and subjected to trimming, adapter removal and removal of short reads using BBDuk within BBTools package. Paired-end reads were aligned to HBoV1 isolate HZ1403 (acc. No. KP710213), HBoV2 strain W153 (acc. No. EU082213), HBoV3 strain W471 (acc. No. EU918736) and HBoV4 strain HBoV4-NI-385 (acc. No. NC_012729), using Bowtie2 v2.4.2 [15]. Samtools v1.12 [16] was used for further processing of alignments and consensus calling. Lower coverage alignments were de novo assembled using Velvet [17] and contigs were searched against database with BLAST to further improve alignments (see below).

### 2.5. Phylogenetic Analysis

Nucleotide sequences of HBoV1 strains were obtained from the GenBank and used to construct alignments and phylogenetic trees. As outgroups, sequences belonging to genotypes HBoV2, HBoV3 and HBoV4, previously described in the literature, were included in the analysis. Alignments were performed using MAFFT [18] and were manually edited with AliView [19]. Phylogenetic trees were generated using the maximum likelihood method with Molecular Evolutionary Genetics Analysis (MEGA) software v6.06. [20], under the most appropriate model of nt substitution determined with jModeltest v2.1.4 [21]. Bootstrap probabilities for 1000 iterations were calculated to evaluate confidence estimates.

The consensus sequences of HBoV strains obtained in this study were deposited in GenBank under acc. Nos. MZ468522-MZ468550.

### 2.6. Evolutionary Rate and Recombination Analysis

Evolutionary rates were estimated using a Bayesian Markov Chain Monte Carlo (MCMC) approach implemented in Beast v1.8.2. [22], with the best fit model selected with jModeltest 2.1.4. Evolutionary rates were calculated using the relaxed (lognormal uncorrelated) molecular clock model. Samples were run for at least 10 million MCMC chains, with sampling every 1000 generations. Convergence was assessed based on the effective sample size using Tracer v1.5. after a 10% burn-in, and only values above 200 were accepted. The uncertainty of the estimates was indicated by 95% highest posterior density (HPD) intervals.

Recombination events were assessed using Recombination Detection Program v4 [23] with different recombination analyses methods (RDP, Bootscan, MaxChi, Chimaera, Siscan and 3Seq). Only full coding regions were considered for recombination analysis.

## 3. Results

### 3.1. Prevalence and Epidemiology of HBoV Infection

Between 1 May 2017, and March 31, 2021, in a total of 957 children tested for 15 respiratory viruses, HBoV was detected in 73 (7.6%) patients, following RV (343; 35.8%), RSV (162; 16.9%), AdV (145; 13.9%), and PIV (101; 10.6). Out of 73 HBoV positive patients in 13 (17.8%) of them HBoV was detected as monoinfection, and in 60 (82.2%) patients in co-detection with one or more respiratory viruses. Co-detection was most frequently seen with HRV (29 patients) and AdV (19 patients).

**Figure 1:**
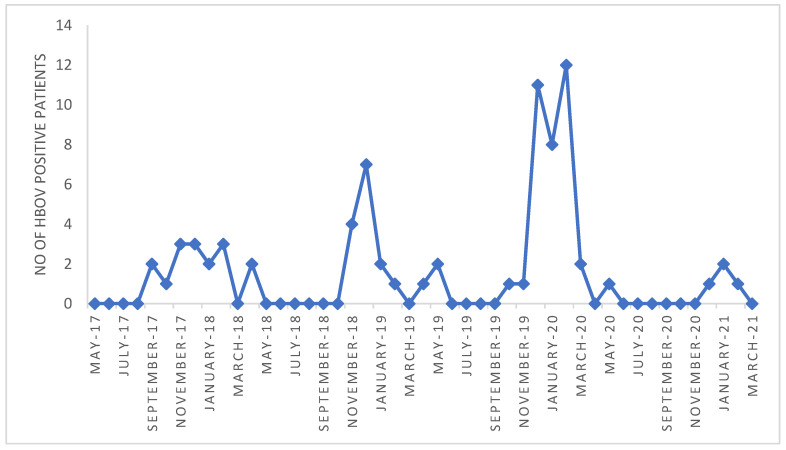
Temporal distribution of the human bocavirus (HBoV) detected (*n* = 73) between May 2017 and March 2021.

The male:female ratio of HBoV positive patients was 41:32 (1.28:1). The median age of HBoV positive patients was 1.36 year (range 7 days to 15 years). According to the age groups, there were 18 (24.6%) patients in the group <1 years of age, 45 (61.6%) patients from 1–2.99 years of age, 6 (8.2%) patients from 3–4.99 years, and 4 (5.5%) patients with ≥5 years of age. With clinical diagnosis of URTI there were 39 (53.4%), and with LRTI 34 (46.6%) patients. Crude demographic data and site of infection of bocavirus infected children were presented in Supplementary File 2. Temporal distribution of detected HBoVs is presented in Figure 1.

### 3.2. Sequencing Results

Out of 73 HBoV positive samples, 49 (67.1%) samples were available for further analysis, of which 29 sequences could be successfully determined. We first started with the approach to amplify near-complete genomes in one PCR amplicon, which was successful in only five samples (HR1119-19, HR1097-19, HR1223-20, HR142-17 and HR387-18), thus a majority of samples had to be amplified in three overlapping fragments. Out of 49 samples subjected to PCR amplification, 24 samples produced amplicons that cover nearly full HBoV genome and 5 produced amplicons covering two-thirds of the genome. As expected, all samples mapped to HBoV1 reference sequence (acc. no. KP710213). In general, samples had high coverage (an average of 13,048×) (Table 1). Four samples (HR1223-20, HR142-17, HR387-18, HR421-18) had much lower coverage and were additionally analyzed by de novo assembly and running BLAST search for contigs over 400 bp. For all samples, longer contigs mainly contained human sequences (not shown); therefore, assembly could not be improved. Despite lower coverage, we were able to retrieve consensus sequence covering whole amplified region, as the lowest mean coverage of 25× was seen in HR142-17 (Table 1).

### 3.3. Phylogenetic and Diversity Analysis

**Figure 2:**
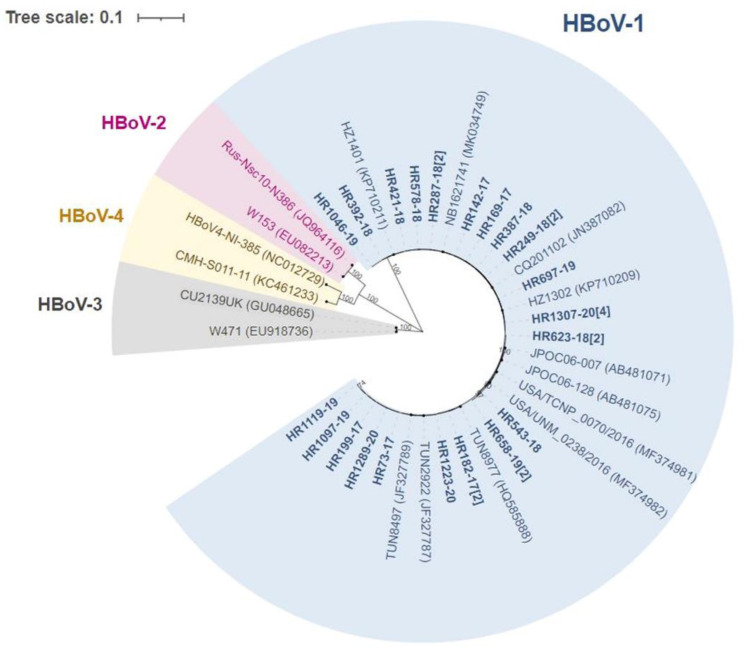
The evolutionary history was inferred by using the maximum likelihood method and general time reversible model. A discrete gamma distribution was used to model evolutionary rate differences among sites. The scale bar indicates the proportion of nucleotide substitutions; the numbers are bootstrap values determined for 1000 iterations (only values above 70% are shown). Nucleotide sequences of HBoV1 strains were obtained from the GenBank and used to construct alignments and phylogenetic trees. As outgroups, sequences belonging to HBoV2, HBoV3 and HBoV4 previously described in the literature were included in the analysis. Croatian sequences have the prefix HR and are indicated in bold; only sequences with unique nucleotide residues were used for phylogenetic analysis, while the numbers in brackets indicate number of strains with identical sequences detected in this study.

The phylogenetic tree was based on 5212 bp of Croatian sequences, along with related sequences retrieved from the database. The tree shows that Croatian HBoV1 sequences are closely related to strains isolated worldwide (e.g., Tunisia, Japan, China, The Netherlands and USA as shown in Figure 2).

There was no phylogenetic grouping based on mono- or co-infection cases or year of isolation. It is also noticeable that sequences are closely related, with only several nucleotide differences and no phylogenetic lineages that can be distinguished with certainty. This was confirmed both at the nucleotide and amino acid level. The identities of our samples ranged from 97.7–99.2 when coding regions were aligned, showing that coding region of *VP3* was least conserved (Table 2). The identities were higher at the protein level: NP1 was fully conserved, while NS1, the longest of NS proteins, had five variable sites. Two of these substitutions are shared with the shorter NS1-70 protein, which lacks the C-terminus: variation at sites 580 (between Asp and Asn) and 627 (Ala and Thr), respectively. NS1 had three additional variable sites in the C-terminal part not shared with NS1-70 (Asn647Asp, Pro668Ser/Thr and Thr722Ala).

Among VP proteins, VP3 had only two variable sites, at 305 (Ser and Asn) and 461 (Ser and Thr), respectively. VP1 had an additional variable site outside of the region shared with VP3, at position 17 where Arg and Lys were observed (not shown). The middle VP protein, VP2 uses a non-canonical start codon [24] and was not analyzed separately since its sequence is already covered by analysis of VP1 and VP3.

Only full coding regions were considered for estimation of rates of evolution. For VP1 and VP3 proteins, this included the shared part as well. Calculated rates of evolution show that HBoV1 strains evolve in the range of 10^−4^ and 10^−5^ substitutions per site and year: *NS1*, *NP1*, *VP1* and *VP3* had a similar mean rate of evolution, while *NS1-70* gene evolves at the rate that resembles complete genome (Table 2). Recombination was not detected among sequences from this study.

## 4. Discussion

This study was performed on 957 Croatian children hospitalized with RTI during period of four years revealed prevalence of HBoV of 7.6%. Furthermore, HBoV was the fifth most frequently detected virus in respiratory tract samples after RV, RSV, AdV and PIV. Previously published retrospective study from Croatia revealed a high detection rate of HBoV among infants and small children with LRTI that required hospitalization (i.e., 23.1% of those with proven viral etiology) [25]. The higher frequency detection of HBoV in comparison to this study may be explained by different designs of the studies, specifically the subject’s age. Namely, the mentioned study included small children up to three years, while this study was performed on patients up to 18 years of age. High HBoV detection in small children is also corroborated by the results of this study; 86.2% of HBoV positive children were younger than three years of age. Moreover, the most recent meta-analysis indicated that being <5 years old is a risk factor for HBoV infection [26]. The same meta-analysis that included 35 studies involving 32,656 subjects from 16 European countries showed that HBoV prevalence varied from 2.0% to 45.69% with a pooled estimate rate of 9.57% [26]. Another review that included 311 studies from 50 countries all over the world performed between 2005 and 2016, showed the average prevalence of HBoV in respiratory tract samples ranged from 1.0% to 56.8%, depending on the country, with the worldwide HBoV total prevalence estimates of 6.3% [27], which is consistent with our results. Furthermore, the same study reported the rate of co-infections in subjects with respiratory infections, and HBoV-positivity ranged from 8.3% to 100%, with total co-infection estimates in the 193 studies covered of 52.4% [27]. In our study, HBoV was co-detected with another respiratory virus in 82.2% cases. High co-detection is a well-recognized characteristic of bocavirus infection, which is probably result of prolonged shedding of HBoV1 to the nasopharynx, including for weeks and months [28]. Furthermore, prolonged shedding of HBoV complicates the diagnosis of acute HBoV infection, thus for accurate diagnosis quantitative PCR, serology, or HBoV1, mRNA detection is recommended diagnostic approaches [29,30]. This also affects the seasonal distribution studies since the actual HBoV1 infection may have occurred months before the current sampling during a later RTI episode.

Most HBoV cases in this study were detected during winter months, from November to February. The northwest part of Croatia, where the study was conducted, has a temperate climate region. Observed seasonal distribution is similar to the prevalence previously reported for temperate regions, where HBoV1 infection mostly occurs in winter and spring, but different from the HBoV1 epidemics in in subtropical regions [31].

Sequencing and phylogenetic analysis shows all samples detected during this study belong to HBoV1, which is in agreement with other studies showing that HBoV1 is primarily a respiratory pathogen, while other HBoVs are prevalent in gastrointestinal infections [32,33,34]. Studies have shown that HBoV2-4 can be detected in respiratory samples at rates of 0.4–4.3%; albeit, their role in respiratory illness remains inconclusive [35,36]. Nevertheless, we have considered this in the sequence assembly step: all samples best assembled to HBoV-1 reference. There was no phylogenetic grouping based on year of isolation.

Out of 29 sequenced samples, amplification of nearly complete genome in one reaction was successful for five samples, unrelated to whether HBoV was detected as single infecting virus or infection with multiple respiratory viruses was detected. It was difficult to obtain long PCR products because of complexity and quality of clinical specimens (nasopharyngeal and pharyngeal flocked swabs combined) although our primers targeted conserved regions at positions which allowed us to divide the genome in three parts. This was most evident in four samples (HR1223-20, HR142-17, HR387-18, HR421-18), which had much lower coverage. BLAST search of these samples revealed that these reads contain primarily human DNA. Nevertheless, with the approach to divide the genome in three smaller segments, we were able to sequence 24 samples and additional five samples contained two out of three genome segments.

Analysis of nucleotide differences show limited heterogeneity between samples and only small differences were observed between different genes. In agreement with previous studies [37,38], genes encoding non-structural proteins are more conserved than VP proteins, which bind to the surface cell receptors and are responsible for transporting the genome to the nucleus [27]. Moreover, VP3 protein (previously called VP2) is the major antigenic determinant [39]; thus, it is expected to be more variable given that mutation and recombination are major drivers of viral evolution [11].

Of the five substitutions in NS1 protein found in our samples, two are located in the middle helicase domain [9] but fall outside of four conserved Walker motifs which execute 3′–5′ helicase function [9,40]. Three substitutions are located in the C-terminal part of NS1 protein, which is predicted to have transcription transactivation capability, but has not been studied [9,41].

VP1 unique region (VP1u) contains phospholipase A2 (PLA2) domain between aa residues 11–66 [42]. Within our samples, a substitution at amino acid residue 17 between Arg and Lys was observed. Most of our samples have arginine at this position, while two samples (HR199-17 and HR1289-20) have lysine. Phospholipase activity of this domain is required for endosomal escape [43], but since both Lys and Arg have positively charged side chains, we do not believe it could have an impact on these processes.

Among our samples, we also observed a change at residue 461 within variable region VR-VIIIB, which is reported to be important for contact with Fab [44]. The impact of this substitution was not examined. However, VR-VIIIB was previously identified as a potential target for the development of a peptide vaccine that would be broadly neutralizing against multiple HBoV strains [44]. Nevertheless, further research that includes available HBoV1 cell culture systems are required to find whether the substitutions and variable sites detected in the sequenced strains had an effect on virus production, infectivity or reactivity with neutralizing anti-HBoV1 antibodies.

Calculated nucleotide based evolutionary rates are consistent with previous studies all showing that HBoV evolves at rates of about 10^−4^ to 10^−5^ substitutions per site and year [37,38]. Slowest evolving was gene encoding NS1-70 protein, which was also the most conserved based on differences analyzed at the nucleotide level. The NP1 protein had a faster rate of evolution, although amino acid analysis shows this protein is completely conserved among our samples, indicating that synonymous substitutions play main role in the evolution of this gene. A study by Lu et al. also shows a slightly higher substitution rate of *NP1* gene [37].

Intra-genotypes recombination appears to play a significant role in the evolution of bocaparvovirus [12]. Using comparison of genome organization and phylogenetic analysis, it was shown that HBoV3 *NS1* and *NP1* sequences cluster with the homologous sequences of the HBoV1 strain, but conversely, the *VP1/VP2* sequences of HBoV3 are similar to HBoV2, providing evidence that HBoV3 may have resulted from recombination between the HBoV1 and HBoV2 viruses [27]. A study from Thailand, using full-length sequence analysis, revealed that an unusual strain of HBoV4 was the result of recombination between HBoV2 and HBoV4 strains [11]. Furthermore, whole genome sequencing of Novosibirsk HBoV isolates detected an isolate that emerged via recombination between HBoV3 and HBoV4 [45]. Although HBoVs are considered to be diverse and frequently recombinant pathogens, especially HBoV2-4 that are primarily replicate in the gastrointestinal tract, recombination was not detected in the samples investigated in this study.

In conclusion, the prevalence of HBoV found in this study are consistent with previously published data confirming HBoV1 as the dominant human bocavirus that causes respiratory infections. Nevertheless, further molecular studies are needed to continuously monitor the evolution of human bocaviruses.

## Figures and Tables

**Table 1:** Summary of sequencing results.

Sample Name	No. of Reads	Reads after QC	% Mapped Reads	Mean Coverage	Length of the Genome Covered **
HR1119-19	923,440	900,168	74.9	12,703	1–5212
HR73-17	1,049,712	893,292	72.8	12,325	1–5212
HR88-17	852,090	822,068	87.4	17,225	1–5212
HR165-17	791,812	764,284	89.3	16,206	1–5212
HR169-17	1,065,688	1,044,282	66.4	14,164	1–5212
HR199-17	1,023,004	997,492	86.1	16,351	1–5212
HR249-18	821,676	772,300	88.2	14,987	1–5212
HR392-18	1,112,498	1,106,784	82.9	34,300 *	1–3303
HR538-18	938,192	929,582	63.6	12,392	1–5212
HR543-18	880,722	850,502	85.6	25,429 *	1515–5212
HR547-18	1,554,628	1,533,056	84.8	25,318	1–5212
HR623-18	199,334	198,980	86.2	4472	1–5212
HR658-19	1,087,006	1,067,550	88.8	23,111	1–5212
HR963-19	951,628	949,956	81.3	17,782	1–5212
HR1046-19	1,016,564	1,012,774	76.1	28,834 *	1–3303
HR1097-19	696,036	637,982	79.6	12,885	1–5212
HR1223-20	693,162	624,032	0.7	118	1–5212
HR1289-20	805,228	797,680	89.0	17,869	1–5212
HR1304-20	785,420	764,868	83.1	15,483	1–5212
HR1307-20	367,584	366,012	80.7	6187	1–5212
HR1340-20	436,744	436,012	75.8	7853	1–5212
HR1350-20	991,316	988,222	83.2	17,847	1–5212
HR142-17	716,450	713,238	0.2	25	1–5212
HR182-17	837,682	815,558	86.6	17,498	1–5212
HR287-18	757,522	735,720	89.7	16,613	1–5212
HR387-18	704,188	678,748	1.3	168	1–5212
HR421-18	484,234	481,492	0.5	78 *	1–3303
HR578-18	986,276	978,436	61.6	21,202 *	1–3303
HR697-19	771,762	744,216	77.5	13,567	1–5212

* Coverage is denoted according to partial coverage of the genome. ** Position according to GenBank sequence KP710213.

**Table 2:** Nucleotide identity percentages and rates of substitution of four genes of HBoV1 strains included in this study.

Protein	Nucleotide Identity	Amino Acid Identity	Mean Evolutionary Rate	95% HPD
NS1	99.1%	99.4%	1.07 × 10^−4^	1.0 × 10^−8^ –2.6 × 10^−4^
NS1-70	99.2%	99.7%		7.4 × 10^−9^ –1.3 × 10^−4^
NP1	98.9%	100%	1.60 × 10^−4^	3.6 × 10^−8^ –4.4 × 10^−4^
VP1	97.9%	99.6%	1.09 × 10^−4^	5.9 × 10^−9^ –2.3 × 10^−4^
VP3	97.7%	99.6%	1.18 × 10^−4^	2.3 × 10^−9^ –3.3 × 10^−4^
Complete genome	98.5%	n.d.	9.78 × 10^−5^	1.6 × 10^−8^ –2.6 × 10^−4^

## Data Availability

Data is contained within the article and Supplementary Material.

## Supplementary Materials

The following are available online at https://www.mdpi.com/article/10.3390/v13091728/s1, Supplementary File 1: Primer sequences and PCR conditions, Supplementary File 2: Crude demographic data and clinical diagnosis for bocavirus infected subjects.
