# Supplementary material for: Prevalence and Molecular Characterization of Human Bocavirus Detected in Croatian Children with Respiratory Infection
Source: Viruses. 2021 Aug 31;13(9):1728. doi: 10.3390/v13091728 (PMC8473146; doi:10.3390/v13091728)
Supplement: Supplementary file 1 [file viruses-13-01728-s001.zip › Supplementary file 1.pdf]

Supplementary file 1

Primer sequences and PCR conditions

Primer	Sequence (5' – 3')	Position*	Annealing (°C)
B_fw	GCCGGCAGACATATTGGATT	1 – 20	63
B1_Rev	GCCACCAACAACCGCGTAGAT	1789 – 1809	
B2_Fw	TTACGGGCCTGCYTCAACAG	1515 – 1534	61
B2_rev	CTGGATCCAATAATTCCACCAA	3282 – 3303	
B3_fw	CATGGAAGCAGATGCCTCC	3045 – 3063	63
B_rev	CGGCTAGGTTCGAGACGG	5195 – 5212	

*in relation to GenBank sequence KP710213.

For full genome amplification, primers B_fw and B_rev were used, with 10 µL of isolated DNA. PCR reaction mixtures contained 1× Phusion HF Buffer (NEB), 200 µM dNTP mix, 0.5 µM of each primer, and 1 U of Phusion polymerase (NEB). PCR conditions were: 98 °C for 5 min, 35 cycles of 98 °C/10 s, 63 °C/30 s, 72 °C/5 min, followed by final extension at 72 °C for 10 min.

For amplification of overlapping pairs annealing temperature was adjusted according to table above and 5 µL of isolated DNA was used in reaction. PCR conditions were: 98 °C for 5 min, 35 cycles of 98 °C/10 s, Tann °C/30 s, 72 °C/1 min 30s, followed by final extension at 72 °C for 10 min.
